# A review of MRI (CT)/US fusion imaging in treatment of breast cancer

**DOI:** 10.1007/s10396-023-01316-9

**Published:** 2023-05-25

**Authors:** Junta Sakakibara, Takeshi Nagashima, Hiroshi Fujimoto, Mamoru Takada, Masayuki Ohtsuka

**Affiliations:** Department of General Surgery, Chiba University Graduate School of Medicine, 1-8-1 Inohana, Chuo-ku, Chiba, Chiba 260-8677 Japan

**Keywords:** Breast cancer, Ultrasonography, Magnetic resonance imaging/computed tomography, Fusion, Non-mass enhancement

## Abstract

The ultrasound fusion imaging system is a diagnostic device developed in Japan that utilizes ultrasound and magnetic positioning/navigation. A position sensor with a probe reads spatial location information from a magnetic field generator and by synchronously displaying ultrasound images and magnetic resonance (MR)/computed tomography (CT) images in real time. Lesions that are difficult to observe via ultrasonography alone, such as non-mass enhancement, can be identified. Furthermore, lesions that are difficult to identify with ultrasound alone indicated for MRI-guided biopsy under the National Health Insurance Scheme can be identified using ultrasound fusion technology, thereby enabling tissue biopsy to be performed under ultrasound guidance. Using this ultrasound fusion technology, not only non-mass enhancement but also small lesions that are difficult to identify using ultrasound alone can be detected, thus ensuring that a more accurate preoperative imaging diagnosis is established, and leading to safer, more reassuring examinations and surgical procedures. In this paper, we outline the use of this ultrasound fusion technology and fusion techniques in the treatment of breast cancer.

## Introduction

Even if magnetic resonance imaging (MRI)-detected lesions are found during contrast-enhanced MRI examinations before surgery, it is difficult to identify them at the same site via ultrasound examination [1]. The reliability of ultrasonography tends to depend on the operator. To solve the various problems of ultrasonography (and to ensure objectivity and reproducibility), the fusion of different (or similar) imaging modalities is effective. Fusion imaging technology has already been used in the biopsy of prostate cancer in the urological field [2–6] and in the treatment of hepatocellular carcinoma in the liver field [7–14]. We herein report on an ultrasonographic diagnostic technology using magnetic fields in the field of breast cancer.

## MRI-detected lesions

**Fig. 1:**
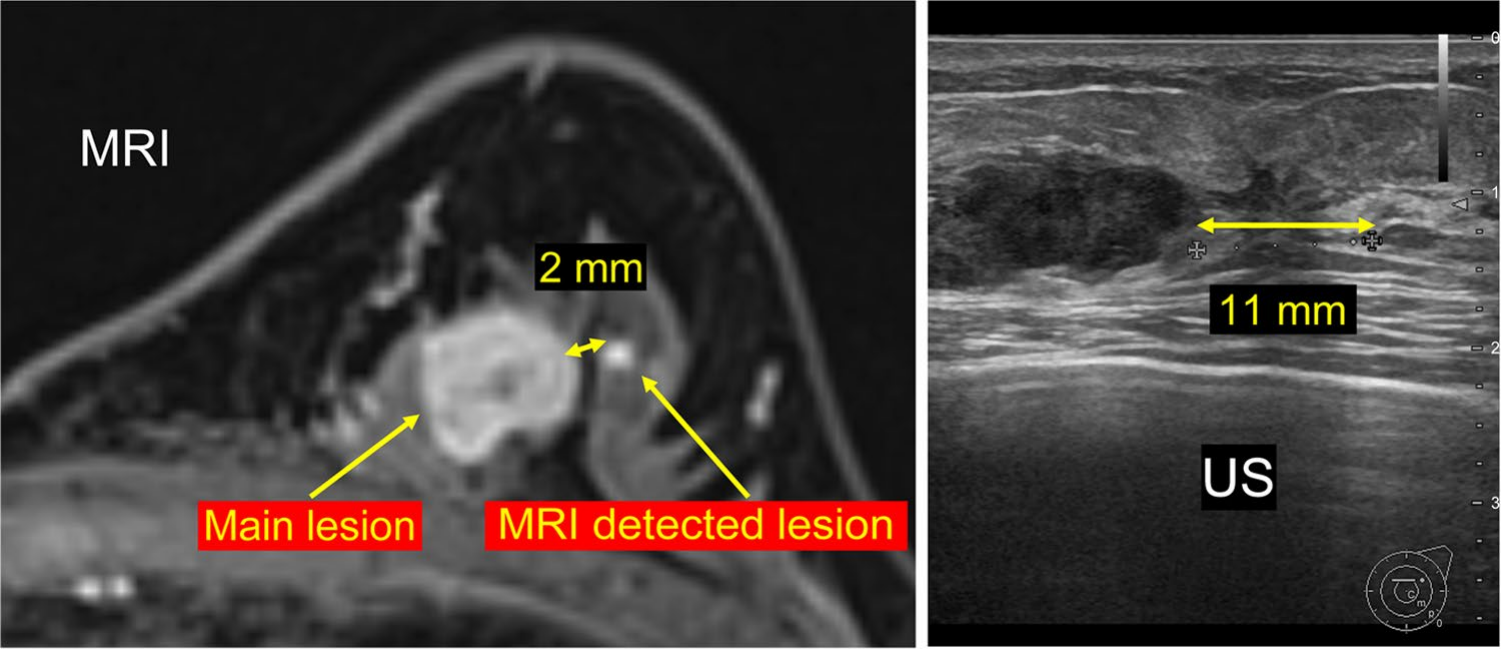
Differences in the spatial relationship of lesions according to patient position. As MRI and ultrasound scans are performed with the patient in different positions (prone and supine, respectively), the spatial relationship of lesions varies between the two imaging modalities. In this figure, the lesions are 2 mm apart on the MRI, but 11 mm apart on the ultrasound image. Owing to such differences, identifying MRI-detected lesions using ultrasound alone is difficult

Ultrasound, which is affordable and places little burden on patients, is the first choice for identifying MRI-detected lesions. However, considering that the patient position of an ultrasound is different from an MRI, it is sometimes difficult to accurately identify MRI-detected lesions using ultrasound. Figure 1 shows how the difference in patient position can change the spatial relationships between lesions. For this reason, the ultrasonographic detection of MRI-detected lesions depends greatly on the skill of the ultrasound technician. Detection rates vary between institutions [15], and ultrasound reportedly has a low identification rate for non-mass enhancement when comparing masses and foci [16].

## Fusion with real-time virtual sonography^®^ (RVS^®^)

**Fig. 2:**
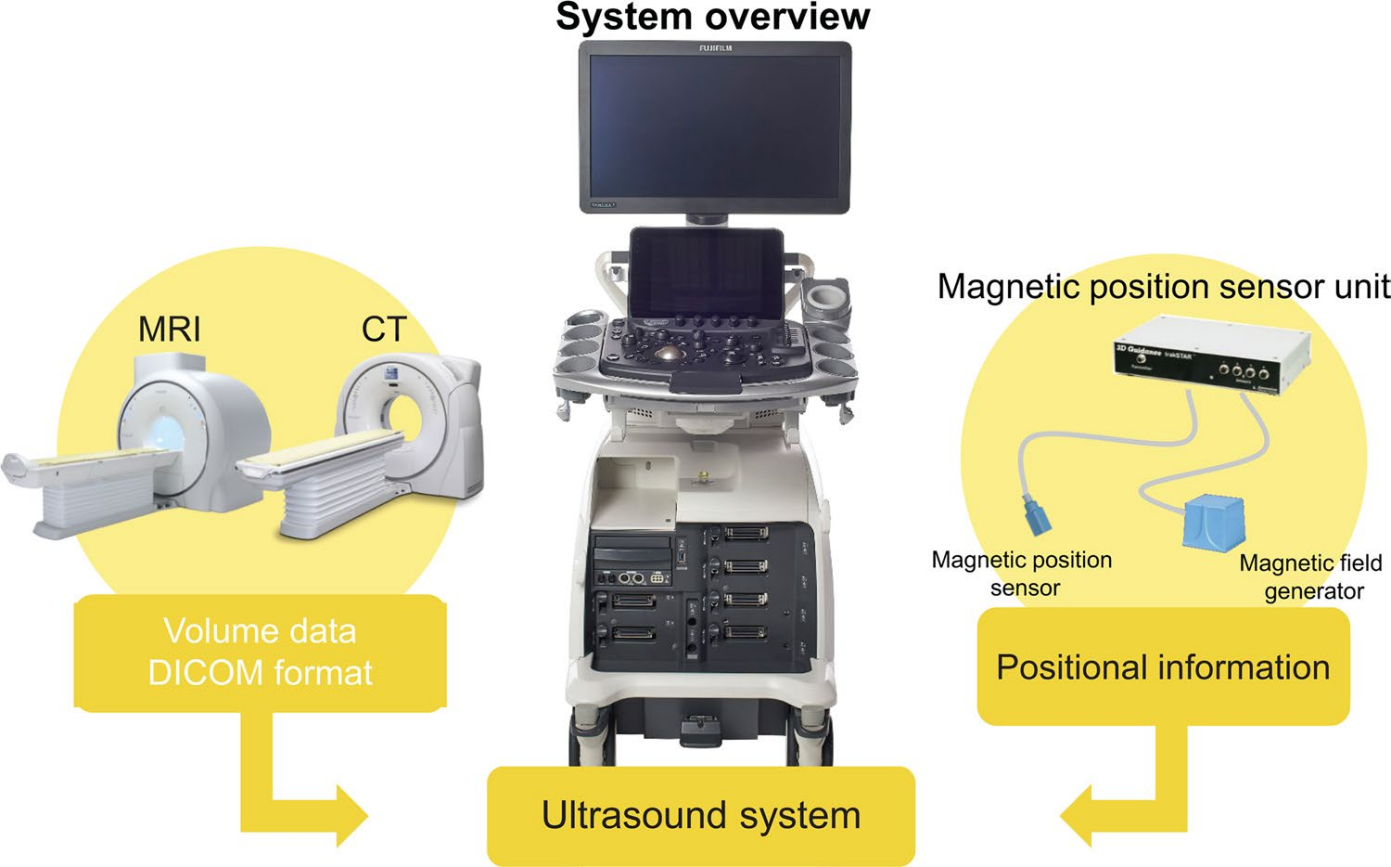
RVS^®^ system overview. Magnetic positional data from the magnetic field generator are detected with a sensor attached to the probe. Feeding volume data (DICOM format) from an MRI or CT scan into the ultrasound system helps clinicians to observe the data alongside ultrasound images in real time

MRI/ultrasound fusion technology is garnering attention as a way to overcome this limitation. The RVS^®^ system is an ultrasound fusion technology with magnetic position navigation developed in Japan by Fujifilm Healthcare. In this diagnostic imaging system (Fig. 2), spatial locational information created by a magnetic field generator is detected by a position sensor attached to an ultrasound probe, and the locational information is used to display MR/CT/ultrasound images together with the ultrasound images from the probe in real time [17–31]. An auxiliary function that links contrast-enhanced MR images and ultrasound images makes it possible to identify MRI-detected lesions that may be difficult to observe with ultrasound alone. This system improves the identification of MRI (or CT)-detected lesions, especially in small tumors, non-mass enhancement, and sentinel lymph nodes [17–32]. In this article, we report on a technique for preoperative diagnosis of breast cancer extent and identification of non-mass enhancement using CT/ultrasound fusion imaging at our institution. Since the fusion system uses magnetic fields during scanning, it is contraindicated for patients with pacemakers as it may cause malfunctions.

## Preoperative diagnosis of breast cancer extent using RVS^®^

**Fig. 3:**
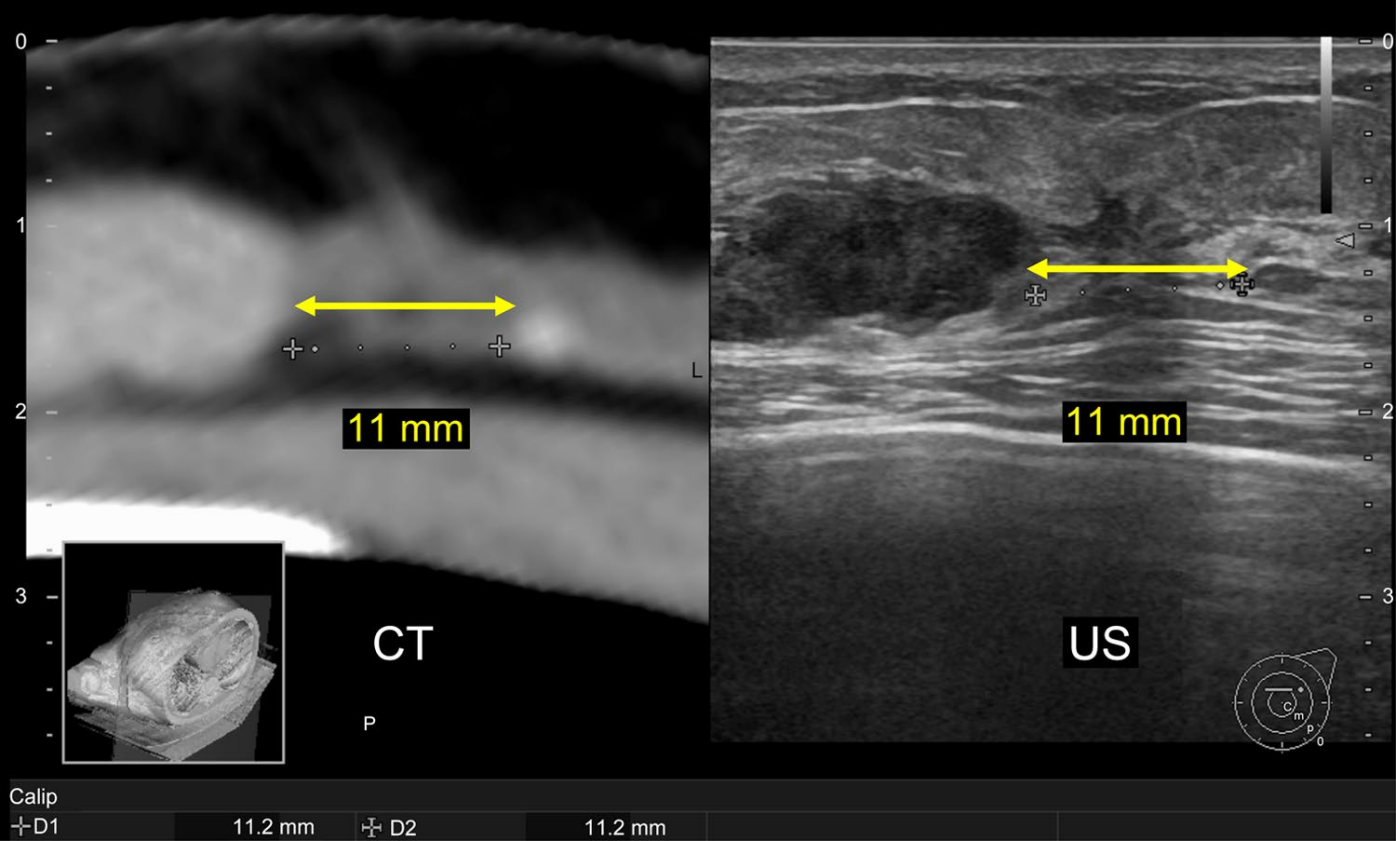
CT/ultrasound fusion image. As contrast CT scans are performed in the same position as ultrasound, the images are easily fused. In this figure, the distance between the lesions in both the CT and ultrasound images is 11 mm

**Fig. 4:**
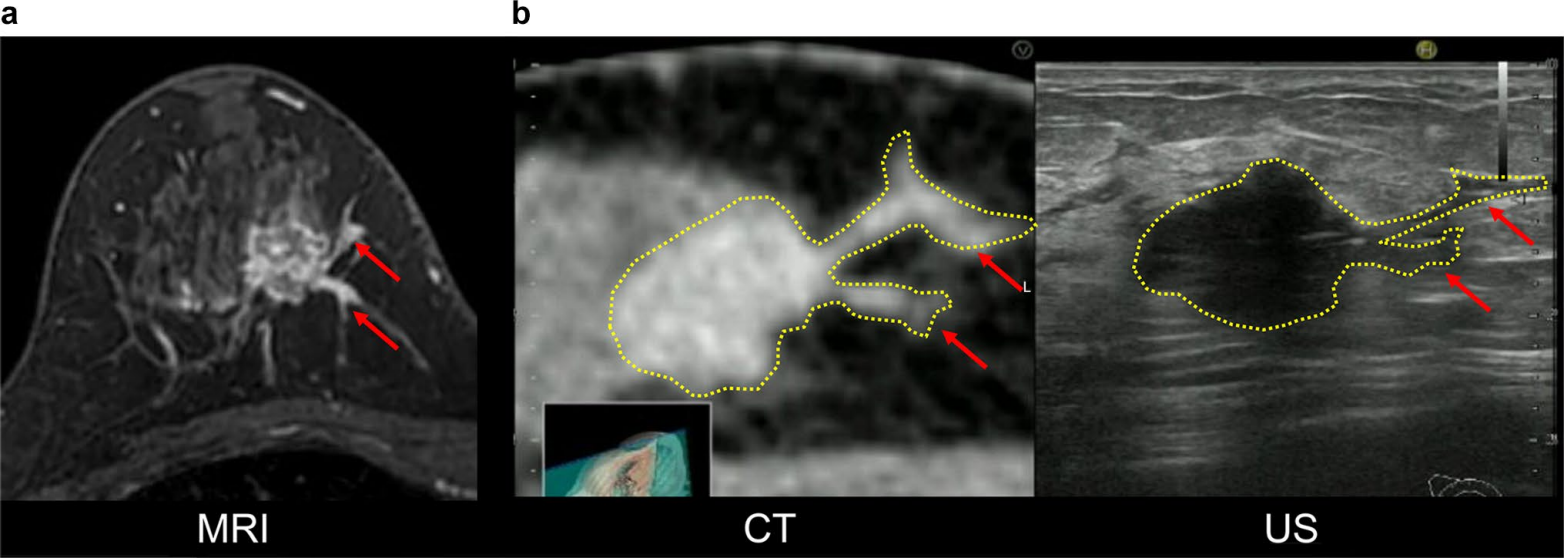
CT/ultrasound fusion image with RVS^®^. **a** Breast cancer lesions and spread presumed to be an intraductal extension (red arrows) were identified in the prone-enhanced MR image. **b** After confirming the lesion corresponding to the MR image on the contrast-enhanced CT image, CT and US fusion using RVS^®^ were performed. Fusion imaging made it possible to identify the extent of the spread of the tumor (red arrows) while ensuring objectivity, which identified the extent of the lesion (yellow dotted line) more accurately

Prone contrast-enhanced MRI is the first step in determining breast cancer extent. If MRI detects additional lesions presumed to be intraductal extension, supine contrast-enhanced MRI is performed in the same position as that for the ultrasound examination. The results, taken together, are useful when conventional ultrasound alone is insufficient for diagnosis. We expect that performing two MRIs poses a tremendous challenge for some institutions, both from a medical standpoint and from the patient’s financial standpoint. In addition, this is a considerable challenge in promoting MRI/ultrasound fusion technology. However, contrast CT is performed in the same position as the ultrasound; thus, it is much easier to combine CT with ultrasound for fusion imaging than performing two MRIs (Fig. 3). At our facility, all breast cancer patients undergo prone-enhanced MRI to diagnose the spread of breast cancer before surgery. In addition, contrast-enhanced CT is also performed to stage breast cancer lesions. For this reason, our facility may have a better environment for fusing ultrasound and CT images than other facilities. Kousaka et al. reported that CT/ultrasound fusion is useful for identifying incidental findings [21]. Breast cancer lesions and spread presumed to be intraductal extension have been identified with prone-enhanced MRI (Fig. 4a). After confirming the lesion corresponding to the MR image in the contrast-enhanced CT image, CT and ultrasound fusion using RVS^®^ was performed (Fig. 4b). First, DICOM data from contrast CT (using GE Revolution^®^, taking 1.25 mm slices) were fed into the ultrasound system. The scan was initiated at the nipple of the affected side. When the probe was moved to the region of the tumor, minor discrepancies may have occurred between the different imaging modalities. To obtain a more accurate fusion, it is important to align the positions of the fat around the lesion, the shape of the mammary gland and ribs, and the blood vessels [27, 28], and to visualize the same cross-section. The CT and ultrasound images were corrected again. In the surgically resected specimen, we pathologically confirmed the intraductal spreading from the tumor, and we believe it matched each image finding.

## Identification of non-mass enhancement using RVS^®^

**Fig. 5:**
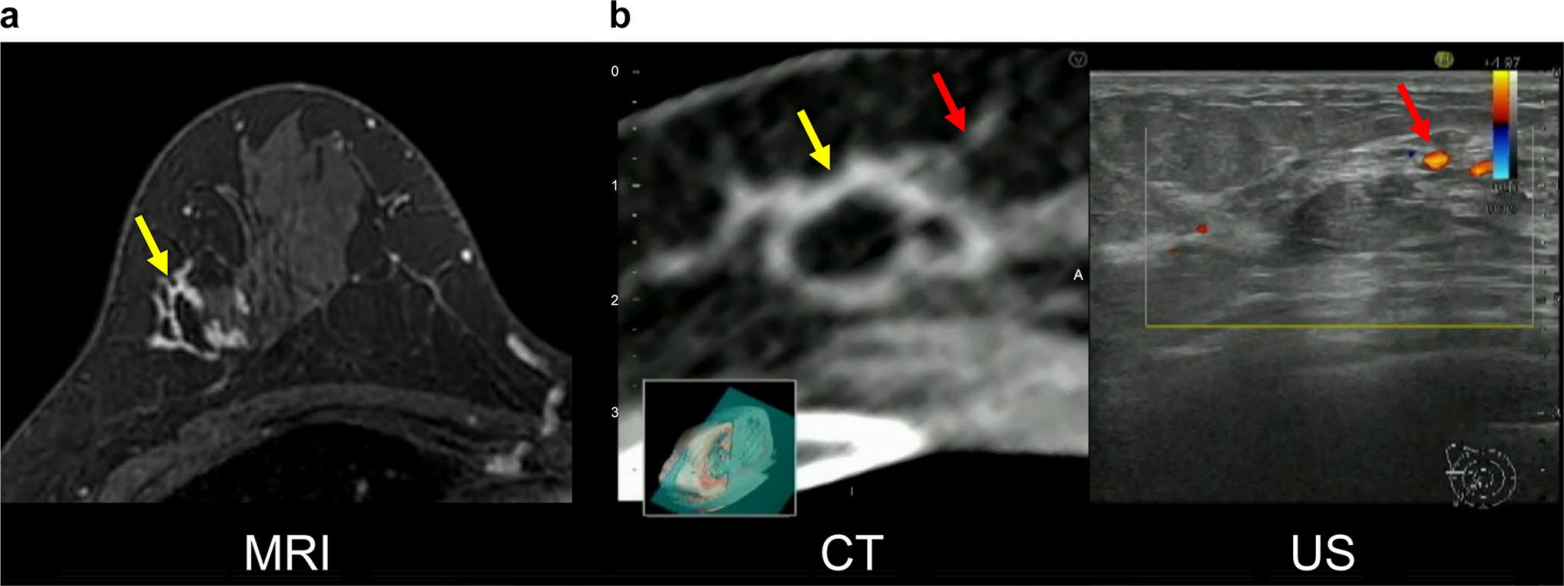
CT/ultrasound fusion image of non-mass enhancement. **a** Non-mass enhancement (yellow arrow) can be seen spreading regionally under MRI. **b** After confirming the region corresponding to non-mass enhancement visualized on the prone position MR image on the contrast-enhanced CT image (yellow arrow), fusion of CT and ultrasound using RVS^®^ was performed. When fusing the images of different modalities, it is important to align the positions of the fat around the lesion, the shape of the mammary gland and ribs, and the blood vessels (red arrow), and to visualize the same cross-section

The technique described above for identifying the extent of intraductal spread from a tumor is a basic technique for RVS^®^. In this section, we describe practical applied techniques to identify non-mass enhancement. In Fig. 5a, non-mass enhancement can be seen spreading regionally under MRI. Under ultrasound alone, identifying the lesion is difficult. RVS^®^ may be particularly effective in such cases. After confirming the area corresponding to non-mass enhancement visualized on the prone position MR image on the contrast-enhanced CT image, fusion of CT and ultrasound using RVS^®^ was performed (Fig. 5b). Our approach is not to immediately begin searching for the lesion but to begin in the anatomical structures surrounding the lesion and working inward to identify it. The site of non-mass enhancement was pathologically confirmed to be ductal carcinoma in situ in the surgically resected specimen.

## Identification of MRI-detected lesions in patients with hereditary breast and ovarian cancer syndrome using RVS^®^

**Fig. 6:**
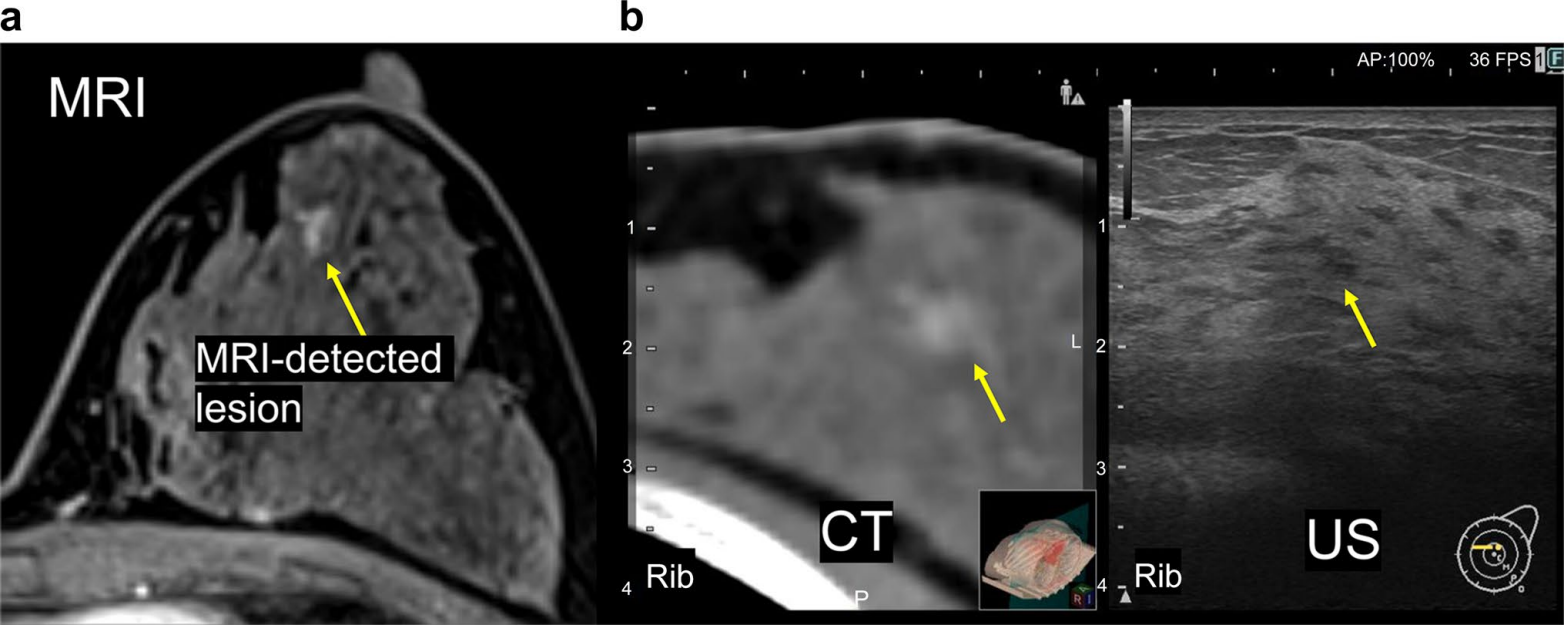
MRI-detected lesion identified with RVS^®^ in a patient with HBOC syndrome. **a** MRI-detected lesion in the unaffected breast of a patient with HBOC syndrome (right breast cancer). **b** After confirming the position of the MRI-detected lesion on the CT image, we fused the CT image with the ultrasound image using RVS^®^. The images created by RVS^®^ clearly identified the MRI-detected lesion (yellow arrow) with improved CT-ultrasonography fusion imaging, which was difficult to perform with ultrasonography alone

In Japan, patients with hereditary breast and ovarian cancer (HBOC) syndrome having breast or ovarian cancer are eligible for surveillance with contrast-enhanced MRI under the National Health Insurance. Although MRI-guided biopsy for MRI-detectable lesions is covered by the insurance, only a limited number of institutions can provide such biopsies. Figure 6a shows the MRI-detected lesion revealed on the unaffected side (left breast) in a patient with HBOC (right breast cancer) syndrome. It was difficult to identify the MRI-detected lesion using ultrasonography alone. Therefore, after confirming the position of the MRI-detected lesion on the CT image, we fused the CT image with the ultrasound image using RVS^®^. As we mentioned earlier, the key to fusion is to use the same sectional view from a CT scan and ultrasonography after registering the position using the shape of fat, a mammary gland, or a rib. Clear identification of the MRI-detected lesion was possible with fusion in this case (Fig. 6b). In this case, instead of tissue biopsy, we used the excised specimen in a risk-reducing mastectomy to search for the MRI-detected lesion. There were no malignant findings, but columnar cell hyperplasia without atypia was observed. As fusion imaging is useful for HBOC syndrome and other cases in identifying non-mass enhancement detected on MRI, its use is expected to increase.

## Lesion identification rate with fusion imaging

In previous studies, the identification rate of MRI-detected lesions was 30–76% [17–19, 22, 28, 30, 33, 34] using conventional ultrasound alone without fusion. In contrast, fusion of supine contrast-enhanced MR images and ultrasound images has been reported to improve identification rates to 78–100% [17–19, 22, 28, 30, 31, 33, 34] (Table 1). In a previous study, a second-look ultrasound was performed on MRI-detected lesions via fusion technology using RVS^®^ or V Nav^®^ (GE Healthcare). After identifying the MRI-detected lesions, a pathological examination was performed under ultrasound guidance. The results showed that 44–77% and 23–56% of lesions were benign and malignant, respectively [18, 22, 28, 30, 31, 34].

**Table 1:** Studies of conventional US and MRI/US fusion for MRI-detected lesions

Authors	Number of MRI-detected lesions	Second-look US (%)	MRI/US fusion (%)
Nakano 2009 [17]	23	7/23 (30)	19/23 (83)
Nakano 2012 [19]	63	42/63 (67)	63/63 (100)
Nakano 2012 [18]	67	18/67 (30)	60/67 (90)
Uematsu 2016 [28]	78	50/78 (64)	24/28 (85.7)
Kang 2017 [30]	119	79/119 (66.4)	31/40 (78)
Watanabe 2017 [22]	59	20/59 (34)	33/39 (85)
Fausto 2019 [34]	722	549/722 (76)	151/173 (87.3)
Goto 2022 [31]	21	–	18/21 (86)

*MRI* magnetic resonance imaging, *US* ultrasound

At our facility, all patients with breast cancer are diagnosed using contrast-enhanced MRI to diagnose the extent of breast cancer before surgery. In addition, contrast-enhanced CT is also performed for staging all breast cancer lesions. If non-mass enhancement is observed on prone-enhanced MR images, an attempt is made to identify the lesion via ordinary second-look ultrasound without using RVS^®^. If non-mass enhancement cannot be identified via second-look ultrasound, after confirming the region corresponding to the non-mass enhancement visualized on the contrast-enhanced MR image in the contrast-enhanced CT image, CT and ultrasound fusion using RVS^®^ is performed.

Kousaka et al. reported a 100% (11/11) rate of identification of the lesion site using fusion of CT and ultrasound images for lesions found incidentally on chest CT [21]. They also reported a breast cancer diagnosis in 36% (4/11) of pathological examinations. The fusion of supine contrast-enhanced MR images and ultrasound images is preferred, but the fusion of contrast-enhanced CT images and ultrasound images may also be clinically useful.

In this manner, a high rate of lesion identification can be achieved using the ultrasound fusion technique for MRI-detected lesions. In the future, MRI-guided biopsies may be indicated for cases that cannot be identified using the ultrasound fusion technique.

## Conclusion

Fusion imaging allows clinicians to observe lesions that cannot be identified with ultrasound alone. This enables appropriate preoperative imaging, and increases the safety of the examinations and surgery. As fusion ensures reproducibility, we think that it is also useful for providing explanations to patients and as an educational tool. We firmly believe that this modality will gain widespread popularity in many clinical settings in the near future.

## Data Availability

Data sharing is not applicable to this article as no new data were created or analyzed in this study.
